# A Genetic-Based Extreme Gradient Boosting Model for Detecting Intrusions in Wireless Sensor Networks

**DOI:** 10.3390/s19204383

**Published:** 2019-10-10

**Authors:** Mnahi Alqahtani, Abdu Gumaei, Hassan Mathkour, Mohamed Maher Ben Ismail

**Affiliations:** Department of Computer Science, College of Computer and Information Sciences, King Saud University, Riyadh 11543, Saudi Arabia; mnahiralqahtani@gmail.com (M.A.); mathkour@ksu.edu.sa (H.M.); mbenismail@ksu.edu.sa (M.M.B.I.)

**Keywords:** intrusion detection system, wireless sensor networks, genetic algorithm, extreme gradient boosting classifier, WSN-DS

## Abstract

An Intrusion detection system is an essential security tool for protecting services and infrastructures of wireless sensor networks from unseen and unpredictable attacks. Few works of machine learning have been proposed for intrusion detection in wireless sensor networks and that have achieved reasonable results. However, these works still need to be more accurate and efficient against imbalanced data problems in network traffic. In this paper, we proposed a new model to detect intrusion attacks based on a genetic algorithm and an extreme gradient boosting (XGBoot) classifier, called GXGBoost model. The latter is a gradient boosting model designed for improving the performance of traditional models to detect minority classes of attacks in the highly imbalanced data traffic of wireless sensor networks. A set of experiments were conducted on wireless sensor network-detection system (WSN-DS) dataset using holdout and 10 fold cross validation techniques. The results of 10 fold cross validation tests revealed that the proposed approach outperformed the state-of-the-art approaches and other ensemble learning classifiers with high detection rates of 98.2%, 92.9%, 98.9%, and 99.5% for flooding, scheduling, grayhole, and blackhole attacks, respectively, in addition to 99.9% for normal traffic.

## 1. Introduction

A wireless sensor network (WSN) is a kind of networks, which can be part of the Internet of Things (IoT) and is composed of a number of sensor nodes. These nodes are distributed in a wide range of different regions to collect required information and convey them to a central node called a base station (BS) node or a sink node, which is a more powerful, capable node [1,2]. They are used in many real-time applications such as security and healthcare monitoring, climate change and environmental monitoring, and military surveillance systems. Several studies have suggested various possible ways to overcome possible security threats related to WSNs. They include secure routing, key exchange, authentication, and other security techniques addressing specific kinds of intrusions. Intrusion detection systems (IDS) are one of the most flexible and useful tools to prevent different attacks and threats to WSNs.

An IDS is an appropriate tool for detecting intrusion attacks in wired and wireless networks. When the system detects the intrusion attack, it alerts the controller or supervisor to take proper decisions [3]. In the last few years, several research works have been published on IDSs for IoT. Some of them are proposed for mobile ad hoc networks (MANETs) [4,5,6]. The others are related to wireless sensor networks (WSNs) [7,8,9], cloud computing [10], and cyber-physical systems [11]. 

Mishra et al. [4] mentioned that the IDSs of wired networks are not an easy to apply for wireless networks because of the difference in their architectures and lack of stable infrastructure. In addition, the authors stated that the responses for detecting the type of intrusion in wireless networks depends on the protocols of network, the confidentiality, the applications, and the heterogeneity in wireless ad hoc networks. These responses may be issued to detect the compromised nodes, reinitializing the network to terminate these nodes, and then sending requests to all nodes in the network for re-authentication. Furthermore, the authors introduce a discussion about seven IDSs proposed for MANETs based on a set of methodologies such as mobile agent-based detection and distributed anomaly-based detection. In the methodology of mobile agent-based detection, the IDS agent on the mobile node can collect local data and perform local detection using mobile agent’s technology. While that the methodology of distributed anomaly-based detection can use the information collected from the neighboring nodes for performing global detection.

Anantvalee and Jie [5] introduced a study about IDS MANETs considering the infrastructure of the network. Based on the nature of MANETs, the authors mentioned that most of the surveyed IDSs could be distributed to have a cooperative structure. As well, this study presents a taxonomy of nodes’ misbehavior in MANETs during detection task, regarding the punishment and route discovery, observation and data distribution, and the architecture and type of data collection. 

Kumar and Dutta [6] presented a review study of intrusion detection techniques in MANETs. The authors in this study focused on the detection methods to classify the intrusion detection techniques based on the mechanisms used in these detection methods. Additionally, the authors stated the challenges that face the IDS in MANETs such as dynamic environments, time of detection, type of attacks, routing protocol, mobility effects, robustness, performance, flexibility, speed, scalability, and reliability.

A taxonomy of IDS for WSNs according to the way that the IDS agent can be used in the network is presented in [7]. In this taxonomy, the IDS agent can be deployed as purely distributed where the IDS is used in each sensor node, or as purely centralized where the IDS is installed in the base-station of the network, and finally as distributed-centralized in which the IDS is deployed in some of monitor nodes. The authors in this study explained the correlation between the position of IDS agent in the WSN and energy consumptions, as well as they mentioned that the IDS of distributed-centralized taxonomy is suitable for WSN with regard to complexity of network’s topology and power consumptions.

Another taxonomy of IDS for WSNs concerning to detection technique that may be anomaly-based detection, misuse-based detection, and specification-based detection is introduced in [8].

Some issues that are investigated in this study include lack of real IDS implementations in WSNs as well as evolving the mechanisms of IDS to deal with the revolution of the IoT. Besides, they presented some research areas of IDS for WSN that need further improvement, such as the tradeoff between consumption of resources and accuracy, the IDS structural design, and the integration between the IDS mechanisms.

An extensive literature review of IDS for WSNs is introduced in Reference [9] and another literature review of IDS for IoT is presented in Reference [12]. In both literature reviews, the authors conclude that some IDSs can be applicable directly, some other IDSs can be applicable with some major modifications, and the rest cannot apply to WSNs due to the requirements of design in the WSNs.

Tsiropoulou et al. [13] described the interference mitigation risk aware (IMRA) problem in the RFID network, which is part of IoT. They formulated the IMRA problem as a non-cooperative game among all normal and intruders tags the RFID network. After that, they proposed a distributed iterative and low-complexity algorithm to solve this problem and maximize the RFID tag’s utility function.

Based on the nature of attacks and the behavior of detection system, there are two kinds of IDS. One of them is known as signature-based IDS. The signature-based IDS can recognize the patterns of well-known intrusion attacks with excellent accuracy, but it is not able to identify new intrusion attacks, which their signatures are not defined in the database of attacks. The other kind is known as anomaly-based IDS that can detect intrusions by identifying the features of intrusion attacks from networks traffics or their resource utilization. In this kind of IDS, several studies are proposed for IDS using a number of machine learning and optimization methods. For example, some of these studies were developed using random forest (RF) [14,15], k-nearest neighbor (KNN) [16], decision tree (DT) [17], particle swarm optimization (PSO) [18], support vector machine [19], genetic algorithm (GA) [20,21,22], and extreme gradient boosting (XGBoost) [23,24,25]. Other studies have been proposed combing SVM with GA [26,27], GA with fuzzy logic (FL) [28,29], GA with deep belief network (DBN) [30], GA with DT [31], and GA with RF [32].

Even though anomaly-based IDS has the capability to recognize both known and unknown attacks, it has some limitations in terms of false negatives and false positives alarms. Similarly, WSNs is not excluded from these intrusion attacks and security threats, which lead to decrease its performance and efficiency. Denial of service (DoS) attacks are the most popular intrusions in WSNs and can be issued in different ways. Each of them uses a specific way of access into the system. For example, there are several different attacks targeting the protocols of WSNs and their layers may lead to DoS [33]. To detect the attacks, network traffic has to be thoroughly analyzed for the purpose of definition of the proper detection technique [34]. This approach uses SVM algorithm to recognize anomalies in the system and creates a signature that would serve for detecting this threatening action in the future [35]. This cluster-based scheme engages detection and avoidance procedures with high-energy efficiency and low overhead of communication [36]. For the localization property, IDS can be employed at various levels of cluster head and sensor nodes. Moon et al. [37] proposed a routing protocol with intrusion detection and prevention at sensor network nodes.

To enhance the system capabilities, an integrated system for intrusion detection at cluster-based of wireless sensor networks has proposed by Wang et al. [38]. Barbancho et al. [39] investigated the usage of artificial intelligence methods in routing schemes of wireless networks to detect intrusion attacks. El Mourabit et al. [40] proposed a method for intrusion detection in wireless sensor networks based on mobile agents. They have used three main mobile agents (collector agent, misuse detection agent, and anomaly detection agent) based on SVM classifier for detection. Shamshirband et al. [41] proposed a competitive clustering algorithm for intrusion detection in WSNs using a density-based fuzzy method. Moreover, Shamshirband et al. [42] proposed an artificial immune system to detect intrusion in WSNs based on cooperative fuzzy theory. In other work, Shamshirband et al. [43] proposed a method to detect sinkhole kind of intrusions. In this method, a number of dubious nodes is produced by a verification process of data consistency and the attacker is recognized by information taken from the data flow.

Kumarage et al. [44] proposed a distributed method for anomaly detection in industrial WSNs using fuzzy data modelling. This distributed method is able to detect the DoS events in which the sink and base-station nodes are used as decision maker players. Sumitha and Kalpana [45] have used a MATLAB programming tool for simulating the DoS attack in WSN using low energy aware cluster hierarchy (LEACH) protocol. In this study, the authors proposed a hybrid method using ant colony optimization with hidden Markov model (ACO + HMM). This hybrid method provides enhanced performance than other methods.

Almomani et al. [46] published a new dataset of different DoS attacks in WSNs, namely, WSN-DS. This dataset consists of four types of DoS attacks (flooding, grayhole, blackhole and scheduling attacks), as well as the normal traffic class. It is created based on LEACH protocol, which is a hierarchical routing protocol in WSNs, and using NS-2 network simulator. A Waikato Environment for Knowledge Analysis (WEKA) data-mining tool was used for implanting neural networks (NNs) to detect the attacks. The results were reported using 10 folds cross-validation and held-out splitting techniques. This study achieved a satisfactory result; however, it suffers from the imbalanced problem in which the detection rate of grayhole attack is very low and reaches up to 75.6%.

Abdullah et al. [47] proposed an approach for detecting intrusions in WSNs’ nodes using a set of machine learning classifiers. These classifiers are SVM, naive Bayesian (NB), DT and RF. Four types of DoS attacks (flooding, grayhole, blackhole, and scheduling attacks) were studied in this work. A WEKA data-mining tool was used for implementing their approach. The results were evaluated based on a number of different metrics, such as recall (R), precision (P), true positive rate (TP), and false positive rate (FP). This study demonstrated that the SVM achieves a high detection rate of 96.7% compared to the other classifiers.

Le et al. [48] proposed to use the random forest (RF) classifier for detecting the type of DoS attacks in WSNs. The proposed classifier attains best F1-score results are 96%, 99%, 98%, 96% and 100% for flooding, blackhole, grayhole, scheduling (TDMA), and normal attacks, respectively. However, the result of this study was for a small number of instances in the testing phase, which approximately represents 25% (94,042 instances) of the data. Recently, Tan et al. [49] proposed a method for intrusion detection using random forest classifier and synthetic minority oversampling (SMOTE) technique. They used the SMOTE technique for oversampling the minority samples. The experimental results of the study showed that the accuracy of using random forest classifier was 92.39% and the accuracy of using SMOTE has increased the accuracy to 92.57%.

## 2. Research Methodology

### 2.1. Genetic Algorithm (GA)

Genetic algorithm (GA) is defined as a heuristic adaptive search algorithm and inspired from the evolutionary ideas of genetics. It represents an intelligent exploitation that uses a random search for solving both unconstrained and constrained optimization problems [50]. The GA repetitively alters individual solutions of a population and at each step, it selects randomly individuals from the population that are currently in process to be parents; then, it utilizes them to generate the children for the next generation of population. Undergoing development of these consecutive generations; the solution is improved to optimality. Genetic algorithm is used to solve a variety of problems, including *mixed integer programming* problems or the problems in which their objective function is *stochastic*, *non-differentiable*, *discontinuous*, or *highly nonlinear*. Generally, the GA applies three different rules on the current population at each step to produce the next generation. These rules are:

- *Selection rules*, which selects the individuals to be *parents* for contributing at next generation;
- *Crossover rules*, which combines two parents to generate the children of next generation;
- *Mutation rules*, which changes randomly the individual of children.

The GA differs from a classical derivative-based optimization algorithm (DOA) in two key ways: it creates a population of solutions at each iteration in which the best solution approaches to optimality and uses a random computation for selecting the next population. While, the classical DOA creates a single solution at each iteration in which a sequence of solutions approaches to the optimal situation and uses a deterministic computation for selecting the next solution in the sequence. Algorithm 1 illustrates the pseudocode of GA as sequence of steps.


**Algorithm 1. The GA pseudocode.**

**Input:** GA parameters
**Begin**
P←Generate-Initial-Population ();Best-Solution ←Evaluate-Fitness(P)
**while** *stopping_criterion is not reached do*
 **Begin**   Parents←Selection(P)   Children←Crossover (Parents)   Children←Mutation (Children)   Best-Solution←Evaluate-Fitness (Children)   P←P ∪ Children
 **End while**
**End**
**Output:** Best-Solution


### 2.2. Gradient Boosting (GB) Model

Gradient boosting (GB) is an ensemble learning technique, used for classification and regression problems, proposed by Friedman [51,52]. It can produce an effective model consisting of weak learners, usually decision trees. The basic idea of GB is to build and generalize the ensemble model in a stage wise fashion by optimizing an objective arbitrary loss function [53]. The GB technique constructs its model from the previous loss function of negative gradient in an iteration manner. In the ML, minimizing the loss function is an important issue and needs to be optimized. In other words, the loss function represents the difference between the predicted output and the target. A low value of loss function means a high prediction or classification result. When the loss function decreases sequentially and iteratively, the model goes consecutively along a specific direction, which is the Gradient of loss function.

Assuming that the objective of a supervised classification problem is to find an approximation function, $\hat{O}\left(x\right)$ to fit the$\;O\left(x\right)$. Therefore, the approximation function based on a loss function,$\;L\left(y,O\left(x\right)\right)$ is defined as:

(1) $$\hat{O}\left(x\right)=\arg\underset{O\left(x\right)}{\min}L\left(y,O\left(x\right)\right)$$

where O represents the weak learners ($C_{i}\left(x\right)$) with weights ($w_{i}$) in a linear combination; and $\hat{O}$ tries to minimize the loss function of the input vector. Thus, the GB sets a constant function,$\;O_{0}\left(x\right)$ as:

(2) $$\left(x\right)=\arg\underset{w}{\min}\overset{n}{\underset{i=1}{\sum }}L\left(y_{i},w\right)$$

The pseudocode of GB is shown in Algorithm 2.


**Algorithm 2. The GB pseudocode.**

1**Input**: $D=\;\{(x_{1},y_{1}),\;(x_{2},y_{2})\;,\;\ldots ,\;(x_{N},y_{N})\},\;L\left(y,\;O\left(x\right)\right)$2
**Begin**
3Initialize: $O_{0}\left(x\right)=\arg\underset{w}{\min}\sum_{i=1}^{n}L\left(y_{i},w\right)$4
for m=1:M
5
$r_{im}=-\frac{\partial L\left(y_{i},O\left(x_{i}\right)\right)}{\partial O\left(x_{i}\right)}$
6Train weak learner $C_{m}\left(x\right)$ **on training data.**7Calculate $w:\;w_{m}=\arg\underset{w}{\min}\overset{N}{\underset{i=1}{\sum }}L(y_{i},O_{m-1}\left(x_{i}\right)+wC_{m}\left(x_{i}\right))$
8Update: $O_{m}\left(x\right)=O_{m-1}\left(x\right)+w_{m}C_{m}\left(x\right)$9
***End for***
10
**End**
11
**Output:** $\;O_{m}\left(x\right)$



In case decision tree is chosen to be an estimator, gradient boosting will be selected as the appropriate algorithm, which is a better classifier that can be utilized for solving many problems in different fields. Previously, we noted that there are different boosting algorithms. Gradient boosting is considered as the most effective one from these algorithms. Although GB mainly depends on convex loss function, it can use different types of loss functions. Moreover, GB can solve regression and classification problems as well. Concerning classification problems, a log loss function is used to be an objective function to deal with these problems. From a fundamental element point of view, GB uses negative gradient to enhance the results.

#### Extreme Gradient Boosting (XGBoost) Model

In the last decade, data science has gained more interest for different fields in many applications. Currently, many big buzzwords such as big data and artificial intelligence has overwhelmed our lives. Boosting algorithms also have evolved with time. A well-known boosting model, which has achieved a high score for solving classification and prediction problems in many contests of the KAGGLE platform, is the extreme gradient boosting (XGBoost) model.

In fact, XGBoost is a type of GB that provides an innovative tree searching technique [54]. The improved technique has shown good performance in distributed computing and avoidance of overfitting, as well as in solving problems that have data sparsity. More precisely, computation complexity is reduced significantly with automatic learning in the splitting process. To tackle the overfitting problem, XGBoost appends regular terms to the objective function in the learning phase.

XGBoost applies second-order Taylor expansion to the loss function to substitute the first derivative unlike conventional GB, as given in Equation (3) as follow:

(3) $$L=\underset{i}{\sum }l\left(y,O\left(x_{i}\right)\right)+\underset{k}{\sum }\Omega \left(G_{k}\right)$$

where, *l* is the loss function of training and *L* define real loss function for XGBoost algorithm. The rest of the notations are constant as the same as boosting methods. *G* is defined as a weak estimator for decision tree, while *F* denotes for prediction. Additionally, decision trees complexity, Ω(*G_m_*) is aggregated with the first term to form the objective function. Regular term definition, Ω(*G_m_*), is calculated as:

(4) $$\Omega \left(G\right)=wT+\frac{1}{2}\alpha \overset{T}{\underset{j=1}{\sum }}s_{j}^{2})$$

where, *T* is denoting number of decision trees’ leaves. While, *w _j_* ^2^ denotes L2 norm of scores for each leaf. The *γ* is a control threshold to split nodes, and *λ* is a coefficient to reduce overfitting problem [55]. The final equation can be formed as:

(5) $$\begin{array}{l} L^{m}=\overset{N}{\underset{i=1}{\sum }}l(y_{i},O_{i}^{m-1}+G_{m}\left(x_{i}\right))+\Omega \left(G_{m}\right)\\ \approx \overset{N}{\underset{i=1}{\sum }}\left[l(y_{i},O_{i}^{m-1})+g_{i}G_{m}(x_{i})+\frac{1}{2}o_{i}G_{m}^{2}(x_{i})\right]+\Omega \left(G_{m}\right)\;\; \end{array}$$

Finally, in the previous equation, two variables define the first derivative and second derivative of the loss function, which are $g_{i}=\partial_{O^{m-1}}l\left(y_{i},O_{i}^{m-1}\right)\;\mathrm{and}\;o_{i}=\partial_{O^{m-1}}^{2}l\left(y_{i},O_{i}^{m-1}\right)$, respectively.

### 2.3. Proposed Genetic-Based Extreme Gradient Boosting (GXGBoot) Model

The basic idea behind the proposed GXGBoot model is to build an optimization task using genetic algorithm on top of XGBoot classifier to increase the classification accuracy of minority classes without significantly affecting the overall accuracy of other classes. The genetic algorithm generates random values for the XGBoot classifier to form a new decision boundary with a highest genetic fitness value.

**Figure 1:**
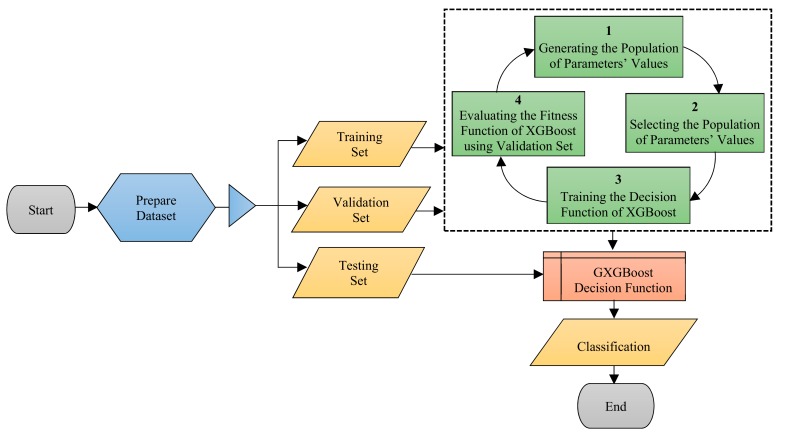
Flowchart of proposed genetic-based extreme gradient boosting (GXGBoot) Model.

More specifically, the GXGBoot model is composed of four main steps: generating the population of parameters’ values, selecting the population of parameters’ values, training the decision function of XGBoot, and evaluating the fitness function of XGBoot. Figure 1 shows the GXGBoot flow chart, and Algorithm 1 outlines the pseudocode of the main steps of GXGBoot.

**Algorithm 3. Pseudocode of GXGBoot’s steps**.
**1.**

**Initialization:**
2.mutation_rate = 0.1 //Mutation rate for GA3.min_mutation_momentum = 0.0001 //Min mutation momentum4.max_mutation_momentum = 0.1 //Max mutation momentum5.min_population = 5 //Min population for GA6.max_population = 10 //Max population for GA7.num_Iterations = 10 //Number of iterations to evaluate GA8.
**Input:**
9.Training Set, Validation Set10.
**Begin**
11.num_population = random.randint (min_population, max_population); // Generate initial population for GXGBoost12.population_GXGBoost = [[]13.For i in range (num_population):14.GXGBoost_parameters = random.randint (min_num_estimators, max_num_estimators) // GXGBoost parameters generation15.GXGBoost_ model = generate_ GXGBoost (GXGBoost_parameters)16.population_GXGBoost.append (GXGBoost_ model)17.End for18.max_accuracy = 019.best_model = None20.population_validation_accuracy= [[] 21.For i in range (num_Iterations):22. For j in range (num_population):23. GXGBoost_model = population_GXGBoost [j] // population selection // population evaluation24. validation_accuracy = evaluate_ GXGBoost (GXGBoost_model, Training_Set, Validation_Set)25.population_validation_accuracy.append (validation_accuracy)26.If validation_accuracy > max_accuracy:27. max_accuracy = validation_accuracy28. best_model = GXGBoost_model29.End if30.End for31.// Create new population with new generations32.# every generation will use the current best GXGBoost_model to mate33.For pop_index in range (num_population):34. model1 = population_GXGBoost [pop_index]35. model1_validation_accuracy = population_validation_accuracy [pop_index]36. model2 = best_model37. model2_validation_accuracy= max_accuracy38. // Create new generation with crossover39. new_model = crossover_GXGBoost (model1, model1_validation_accuracy, model2, model2_validation_accuracy)40. mutate_GXGBoost (new_model) // Mutate new generation41. population_GXGBoost [pop_index] = new_model // Replace current model 42.End for43.End for 44.**Return** best_model, max_accuracy45.
**End**


#### Time Complexity Analysis of Proposed Model’s Algorithm

Based on the computational complexity theory, time complexity analysis is used to compute the computational time of the proposed algorithm. Therefore, the worst case of running time can be defined as a function of its input using big O notation [56]. The big O notation usually defines the asymptotic behavior or the growth rate of the function’s upper bound as follows:

(6) $$O\left(g\left(n\right)\right)=\{f|\exists c\rangle 0,\;\exists n0>0,\;\forall n\geq n0:\;0\leq f\leq cg\left(n\right)\}$$

This means that $f\in \left(g\left(n\right)\right)$ if and only if there exist two positive constants c and n0 for all n≥n0 such that the inequality $0\leq f\leq cg\left(n\right)$ is satisfied. In this case, we can say that f is big O of $g\left(n\right)$ or that $g\left(n\right)$ is the asymptotic upper bound for f [57]. By analyzing the main steps of Algorithm 3, the pseudocode contains a for loop in line 13. This loop depends on the number of populations and contains a generation operation of gradient boosting model with random values for its parameters. The generating operation takes a constant time c. Suppose that the number of populations in worst case is also n. Consequently, this loop runs in$\;O\left(cn\right)$. In addition, the pseudocode contains a for loop in line 21. This for loop depends on the number of iterations and contains two independent for loops. Each one depends on the number of populations. Let us assume that the number of iterations is n and the number of populations is n in the worst case. In line 24, the first for loop contains a construction operation of the gradient boosting model. According to Reference [58], the time complexity to construct the gradient boosting model is$\;O\left(dn\right)$, where d represents the number of features, and n is the number of data samples. Thus, the first for loop runs in$\;O\left(n^{3}\right)$. The second for loop has a set of operations that have a constant time c and runs in$\;O\left(cn\right)$. Therefore, the Algorithm 3 runs in a cubic polynomial time for building the GXGBoot model.

## 3. Experiments and Discussion

### 3.1. WSN-DS Dataset

**Figure 2:**
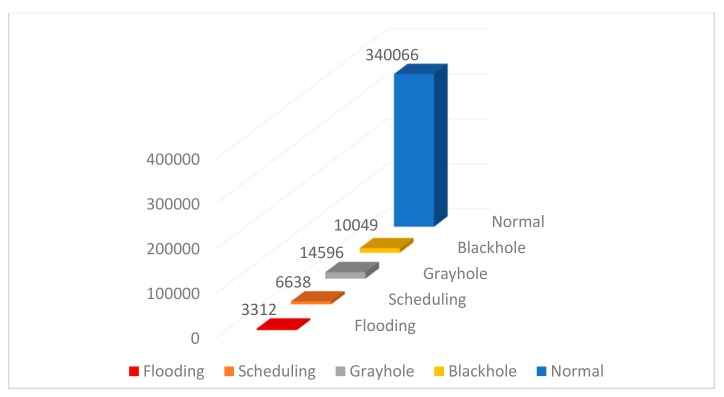
Distribution of attacks in the WSN-DS Dataset.

In our experiments, a simulated WSN-DS dataset collected by Almomani et al. [46] is used as a case study to evaluate the proposed model. This dataset was generated to apply machine-learning methods for detecting and classifying Denial of Service (DoS) attacks. By using machine-learning methods, the sensor nodes can be able to detect attacks patterns from the normal traffic. As a result, the sensor nodes can make a right decision instantly on time. The simulated dataset contains 23 features extracted using LEACH routing protocol as shown in Table 1. The Low Energy Aware Cluster Hierarchy (LEACH) is a routing protocol which uses 23 features to identify the state of each sensor node in the wireless network. However, only 19 features as well as the class label were included in the dataset file. These 19 features were *Id, Time, Is_CH, who_CH, Dist_To_CH, ADV_S, ADV_R, JOIN_S, JOIN_R, ADV_SCH_S, ADV_SCH_R, Rank, DATA_S, DATA_R, Data_Sent_BS, Dist_CH_BS, Send_code, Consumed_Energy,* and *Attack_Type*. The distribution of attacks in the WSN-DS dataset is given in Figure 2. Furthermore, a number of data samples from this dataset is listed in Table 2.

To prepare training and testing sets, the holdout method is used to separate the dataset into 60% training and 40% testing. The number of instances in these two sets is presented in Table 3.

### 3.2. Evaluation Metrics

A set of evaluation metrics including the accuracy (ACC), precision (PR), recall (RE), and f1-score are utilized to evaluate and compare the results of proposed intrusion detection model. They were used because they produced comparable results and frequently used in the machine learning field for evaluating and comparing its models. These performance evaluation metrics are computed as:

Accuracy (ACC) = (TP + TN) ⁄ (TP + FP + TN + FN)(7)

Precision (PR) or Positive Predictive value (PPV) = TP/(TP + FP)(8)

Recall (RE) (True Positive Rate) (Sensitivity) = TP ⁄ (TP + FN)(9)

F1-Score = 2*((Precision * Recall)/(Precision + Recall))(10)

Specificity, Selectivity or True Negative Rate (TNR) = TN ⁄ (TN + FP)(11)

False Positive Rate (FPR) = FP/(FP + TN)(12)

False Negative Rate (FNR) = FN/(FN + TP)(13)

where TP, TN, FP, and FN are the true positive, true negative, false positive, and false negative, computed from the confusion matrix.

### 3.3. Experimental Results and Comparisons

The subsection describes the experimental results and comparisons with other models and related works. The results of our experiment are obtained using both 10 fold cross validation and holdout methods on the simulated WSN-DS dataset [46]. In the 10 fold cross validation, the dataset is divided into 10 parts, one of them is used for testing for 10 times. Table 4, Table 5, Table 6, Table 7 and Table 8 show the results of the 10 fold cross validation method.

**Figure 3:**
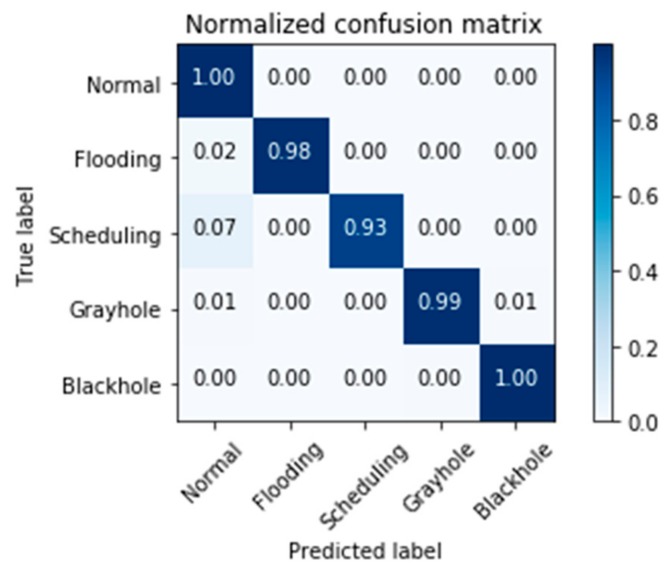
Confusion matrix of intrusion detection of the proposed GXGBoost model using holdout method on the WSN-DS Dataset.

Figure 3 demonstrates the confusion matrix of intrusion detection for the proposed GXGBoost model using holdout method on the WSN-DS Dataset.

Table 9 lists the true positive, true negative, false positive, and false negative rates results of the GXGBoost model using the holdout method. While, the precision, recall, and F1-score results and their weighted average using the holdout method are shown in Table 10. 

### 3.4. Comparison with other Boosting Algorithms

**Figure 4:**
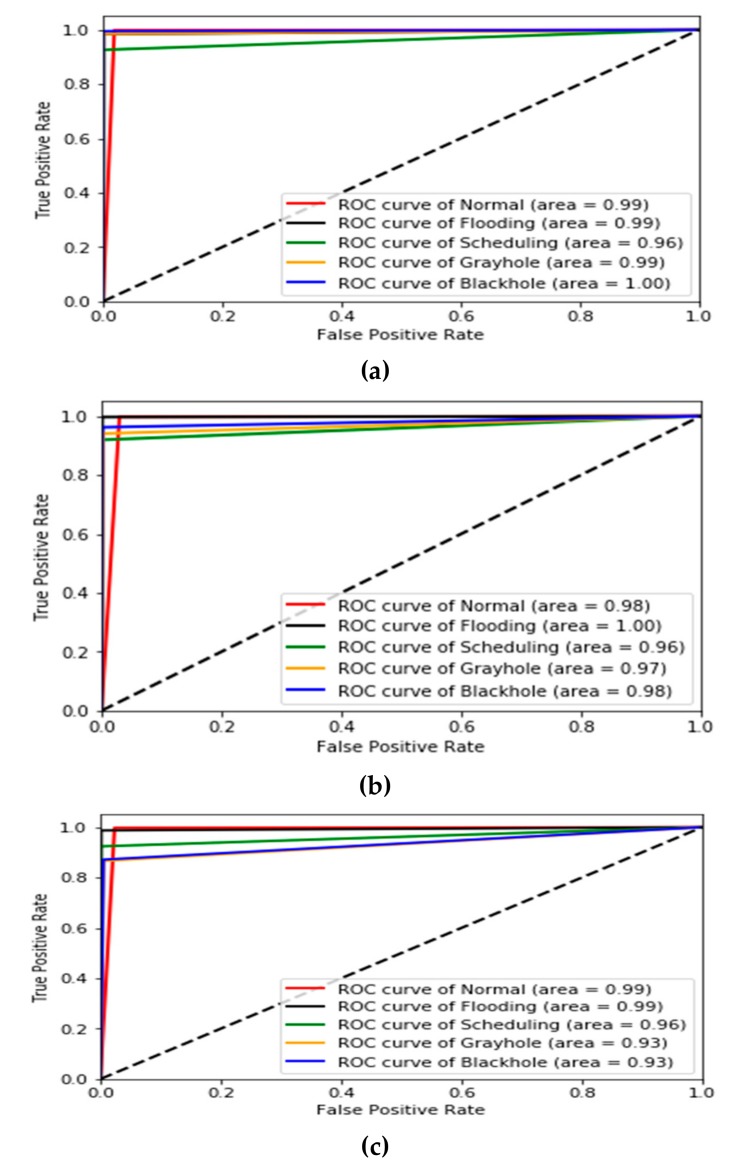

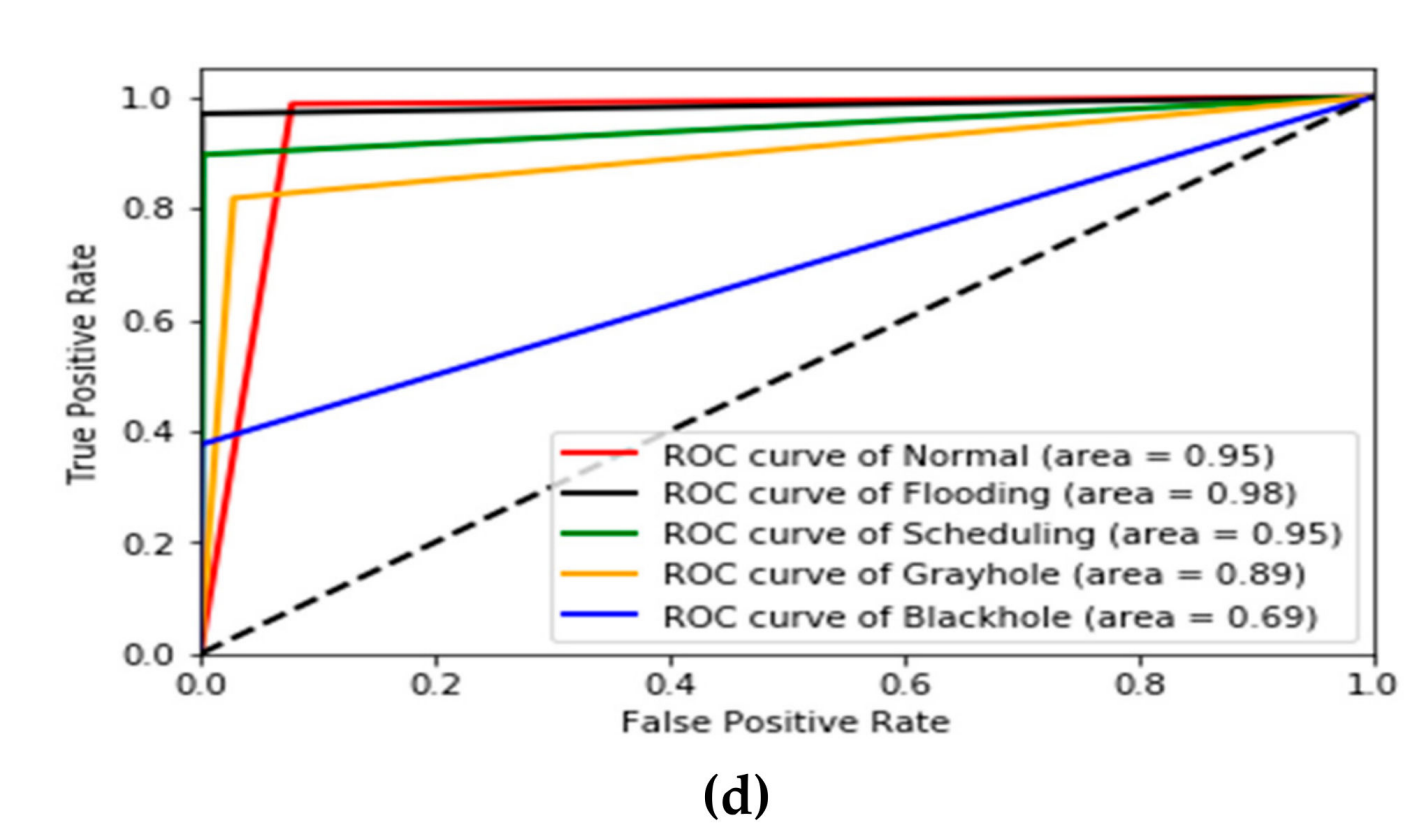
ROC curves for the compared classifiers models: (**a**) ROC curve of GXGBoost, (**b**) ROC curve of XGBoost, (**c**) ROC curve of GB, and (**d**) ROC curve of AdaBoost on the WSN-DS Dataset.

For comparing the GXGBoost model with original XGBoost and other boosting classifiers models such as AdaBoost and gradient boosting (GB) classifiers, we used the true positive rate and receiver operating characteristic (ROC) curve as evaluation metrics to describe their performance. The ROC curve represents the area under curve (AUC) in which when it has a value close to 1, this confirms that the model produces better results. Table 11 and Figure 4 demonstrate the experimental results of the evaluation metrics for the proposed GXGBoost model compared to other boosting models.

To evaluate the efficiency of boosting algorithms for WSNs intrusion detection, the experiments are conducted on a laptop Intel(R) Core(TM) i7-4510U 2.0 GHz and 8 GB RAM with Windows 10. The average execution time of classification for the GXGBoot and other boosting models on the testing dataset is shown in Table 12.

We can see that the classification time of GXGBoost and XGBoost is close to each other. However, the average classification time of GXGBoost is lower than XGBoost since it selects appropriate values for its parameters in the training phase. The AdaBoost has a higher classification time because it tries to classify all the cases into majority classes without losing the overall accuracy. In general, as seen in Table 10, the proposed model is efficient for real-time WSNs intrusion detection.

### 3.5. Comparison with Related Work

**Figure 5:**
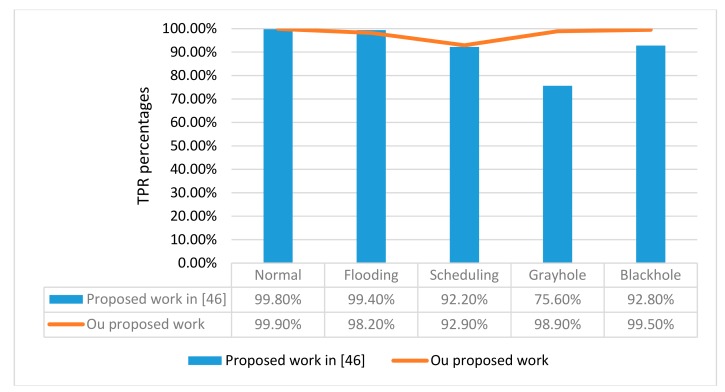
TPR percentage values of the proposed GXGBoost compared to the results of related work in Reference [46] using 10 fold cross validation method.

To compare our work with the related recent work on the same dataset, the true positive rate (TPR) is used as a uniform metric to do that. Figure 5 shows the values of TPR for the proposed GXGBoost compared to the results of related work in Reference [46] using a 10 fold cross validation method.

From Figure 5, we can see how the GXGBoost is effective to classify with the minority classes without significantly affecting the detection rates of the other classes.

## 4. Conclusions and Future work

A new model for WSN intrusion detection is proposed based on genetic algorithm (GA) and extreme gradient boosting (XGBoot) classifier, called GXGBoost model. The GXGBoost model was designed to improve the performance of traditional models to detect minority classes of attacks in highly imbalanced data traffics of wireless sensor networks. A set of experiments were conducted on WSN-DS dataset using holdout and 10-folds cross validation techniques. The results of 10-folds cross validation test revealed that the proposed model outperforms the state-of-the-art models and other ensemble learning classifiers with high detection rates of 98.2%, 92.9%, 98.9%, and 99.5% for Flooding, Scheduling, Grayhole, Blackhole attacks, respectively, in addition to 99.9% for Normal traffic. In the future work, we will apply our model with feature selection methods to reduce the number of features and enhance the efficiency of intrusion detection in WSN.

## Figures and Tables

**Table 1:** Extracted features of the wireless sensor network-detection system (WSN-DS) Dataset.

NO.	Feature Name	Symbol	Description
1	Node ID	Id	It is a unique symbolized number of the sensor node. For example, the sensor node number 13 in the fourth round and in the second stage has ID 002004013.
2	Time	Time	It is the current time of the sensor node state in the simulation.
3	Is CH?	Is_CH	It is a flag, which has 1 or 0 value for determining the node is cluster head (CH), or not.
4	Who CH	who_CH	It is the ID of the cluster head (CH) in the existing round.
5	Received Signal Strength Indication	RSSI	It is the RSSI between a sensor node and its cluster head in the existing round.
6	Distance to cluster head	Dist_To_CH	It is the computed distance between a sensor node and its cluster head in the existing round.
7	Max distance to cluster head	M_D_CH	It is the maximum computed distance between sensor nodes and its cluster head within the same cluster.
8	Average distance to cluster head	A_D_CH	It represents the average distance between sensor nodes within the cluster and their cluster head.
9	Current energy	Current_Energy	It is the current energy of the current round for a sensor node.
10	Energy consumption	Consumed_Energy	It is the energy amount consumed by the sensor node in the previous round.
11	Advertise cluster head sends	ADV_S	It is the number of advertise broadcast messages sent from the cluster head to the sensor nodes.
12	Advertise cluster head receives	ADV_R	It represents the number of advertise messages which are received by the sensor nodes from cluster heads.
13	Join request messages send	JOIN_S	It is the number of join request messages, which are sent by the sensor nodes to the cluster head.
14	Join request messages receive	JOIN_R	It is the number of join request messages, which are received by the cluster head from the sensor nodes.
15	Advertise SCH sends	ADV_SCH_S	It represents the number of advertise broadcast messages of the Time Division Multiple Access (TDMA) schedule which are sent to the sensor nodes.
16	Advertise SCH receives	ADV_SCH_R	It is the number of advertise broadcast messages for the TDMA schedule which are received from cluster heads.
17	Rank	Rank	It represents the order of the sensor node within the schedule of the TDMA.
18	Data sent	Data_S	It represents the number of data packets, which are sent from a sensor node to its cluster head.
19	Data received	Data_R	It represents the number of data packets that are received by a sensor node from cluster head.
20	Data sent to base station	Data_Sent_BS	It represents the number of data packets that are sent from a sensor node to the base station.
21	Distance cluster head to base station	Dist_CH_BS	It represents the distance between the cluster head and the base station.
22	Send Code	Send_code	It is the sending code of the cluster.
23	Attack Type	Attack_Type	It is the class label of the wireless sensor network traffic, which could be normal, or attack. There are four categorical types of attacks, namely, flooding, scheduling (TDMA), grayhole, and blackhole.

**Table 2:** Data samples from the WSN-DS dataset [46].

Id	Time	Is CH	Who CH	Dist To CH	ADV S	ADV R	JOIN S	JOIN R	SCH S	SCH R	Rank	DATA S	DATA R	Data Sent To BS	Dist CH To BS	Send Code	Consumed Energy	Attack Type
101000	50	1	101000	0	1	0	0	25	1	0	0	0	1200	48	130.0854	0	2.4694	Normal
101001	50	0	101044	75.32345	0	4	1	0	0	1	2	38	0	0	0	4	0.06957	Normal
101002	50	0	101010	46.95453	0	4	1	0	0	1	19	41	0	0	0	3	0.06898	Normal
101004	50	0	101010	4.83341	0	4	1	0	0	1	25	41	0	0	0	3	0.06534	Normal
2901024	3553	1	2901024	0	1	9	0	0	0	0	0	0	0	1	113.2765	0	0.01237	Grayhole
2901029	3553	1	2901029	0	1	9	0	0	0	0	0	0	0	1	150.3168	0	0.01237	Grayhole
2901073	3553	1	2901100	0	1	9	0	0	0	0	0	0	0	2	96.57363	0	0.01813	Grayhole
501014	1703	1	501100	0	1	26	0	0	0	0	0	0	0	0	0	0	0.00446	Blackhole
501021	1703	1	501100	0	1	26	0	0	0	0	0	0	0	0	0	0	0.00445	Blackhole
501029	1703	1	501100	0	1	26	0	0	0	0	0	0	0	0	0	0	0.00446	Blackhole
501030	1703	1	501100	0	1	26	0	0	0	0	0	0	0	0	0	0	0.00445	Blackhole
404017	2203	1	404100	0	1	9	0	3	3	0	0	0	0	0	0	0	0.18101	TDMA
404018	2203	0	404028	8.59592	0	10	1	0	0	1	1	160	0	0	0	3	0.26334	Normal
404020	2203	0	404100	12.89353	0	10	1	0	0	1	1	181	0	0	0	4	0.29774	Normal
404023	2203	0	404100	19.59164	0	10	1	0	0	1	1	181	0	0	0	1	0.47633	Normal
404025	2203	1	404100	0	1	9	0	1	1	0	0	0	241	241	138.3672	0	2.02545	TDMA
404028	2203	1	404100	0	1	9	0	4	4	0	0	0	0	0	0	0	0.00623	TDMA
404029	2203	0	404100	18.31869	0	10	1	0	0	1	1	206	0	0	0	5	0.33993	Normal
404035	2203	0	404100	15.82954	0	10	1	0	0	1	1	181	0	0	0	1	0.47308	Normal
404050	2203	1	404100	0	1	9	0	2	2	0	0	0	0	0	0	0	0.00624	TDMA
404053	2203	0	404100	19.42763	0	10	1	0	0	1	1	160	0	0	0	3	0.2652	Normal
404060	2203	1	404100	0	1	9	0	2	2	0	0	0	0	0	0	0	1.09609	TDMA
404073	2203	0	404100	14.13972	0	10	1	0	0	1	1	206	0	0	0	5	0.33878	Normal
404078	2203	0	404100	10.54019	0	10	1	0	0	1	1	206	0	0	0	2	1.42778	Normal
404080	2203	1	404100	0	1	9	0	1	1	0	0	0	241	241	176.6235	0	2.5962	TDMA
302096	1153	1	302096	0	6	22	0	0	0	0	0	0	0	13	121.695	0	0.35722	Flooding
401001	1203	1	401001	0	6	20	0	0	0	0	0	0	0	13	136.2575	0	0.2398	Flooding
401034	1203	1	401034	0	6	24	0	0	0	0	0	0	0	13	165.4621	0	0.26426	Flooding
401054	1203	1	401054	0	6	20	0	0	0	0	0	0	0	13	142.1079	0	0.24251	Flooding
401069	1203	1	401069	0	6	26	0	0	0	0	0	0	0	13	93.93772	0	0.21994	Flooding
101000	50	1	101000	0	1	0	0	25	1	0	0	0	1200	48	130.0854	0	2.4694	Normal
101001	50	0	101044	75.32345	0	4	1	0	0	1	2	38	0	0	0	4	0.06957	Normal
101004	50	0	101010	4.83341	0	4	1	0	0	1	25	41	0	0	0	3	0.06534	Normal
2901024	3553	1	2901024	0	1	9	0	0	0	0	0	0	0	1	113.2765	0	0.01237	Grayhole
2901029	3553	1	2901029	0	1	9	0	0	0	0	0	0	0	1	150.3168	0	0.01237	Grayhole
2901073	3553	1	2901100	0	1	9	0	0	0	0	0	0	0	2	96.57363	0	0.01813	Grayhole
501014	1703	1	501100	0	1	26	0	0	0	0	0	0	0	0	0	0	0.00446	Blackhole
501029	1703	1	501100	0	1	26	0	0	0	0	0	0	0	0	0	0	0.00446	Blackhole
501030	1703	1	501100	0	1	26	0	0	0	0	0	0	0	0	0	0	0.00445	Blackhole
404017	2203	1	404100	0	1	9	0	3	3	0	0	0	0	0	0	0	0.18101	TDMA

**Table 3:** The dataset separated 60% training set and 40% testing set using holdout method.

The Attack Type	Training Set (60%)	Testing Set (40%)
Blackhole	6029	4020
Grayhole	8758	5838
Flooding	1988	1324
Scheduling	3982	2656
Normal	204,039	136,027
Sum	224,796	149,865

**Table 4:** Precision results of the 10 fold cross validation.

Fold No.	Normal	Flooding	Scheduling	Grayhole	Blackhole
1	1.00	0.96	0.99	0.99	0.99
2	1.00	0.97	0.99	0.99	0.99
3	1.00	0.97	0.99	0.99	0.99
4	1.00	0.95	0.99	0.99	0.99
5	1.00	0.94	0.98	0.99	0.99
6	1.00	0.95	0.98	0.99	0.99
7	1.00	0.97	1.00	0.99	0.99
8	1.00	0.94	0.99	0.99	1.00
9	1.00	0.96	0.99	0.99	0.99
10	1.00	0.97	0.99	0.99	0.99

**Table 5:** Recall results of the 10 fold cross validation.

Fold No.	Normal	Flooding	Scheduling	Grayhole	Blackhole
1	1.00	0.99	0.93	0.99	0.99
2	1.00	0.98	0.93	0.99	0.99
3	1.00	0.98	0.93	0.99	1.00
4	1.00	0.99	0.92	0.99	1.00
5	1.00	0.98	0.92	0.99	1.00
6	1.00	0.98	0.94	0.99	0.99
7	1.00	0.98	0.95	0.99	1.00
8	1.00	0.99	0.92	0.99	0.99
9	1.00	0.98	0.93	0.98	0.99
10	1.00	0.98	0.91	0.99	1.00

**Table 6:** F1-score results of the 10 fold cross validation.

Fold No.	Normal	Flooding	Scheduling	Grayhole	Blackhole
1	1.00	0.97	0.96	0.99	0.99
2	1.00	0.97	0.96	0.99	0.99
3	1.00	0.97	0.96	0.99	0.99
4	1.00	0.97	0.95	0.99	0.99
5	1.00	0.96	0.95	0.99	0.99
6	1.00	0.96	0.96	0.99	0.99
7	1.00	0.97	0.97	0.99	1.00
8	1.00	0.97	0.96	0.99	1.00
9	1.00	0.97	0.96	0.99	0.99
10	1.00	0.97	0.95	0.99	1.00

**Table 7:** Positive and negative rates results of the 10 folds cross validation.

	Normal	Flooding	Scheduling	Grayhole	Blackhole
TPR	0.999	0.982	0.929	0.989	0.995
TNR	0.982	1	1	1	1
FPR	0.018	0	0	0.1	0
FNR	0.001	0.018	0.071	0.011	0.005
Overall Accuracy	0.997				

TPR: true positive rate, TNR: true negative rate, FPR: false positive rate, and FNR: false positive rate.

**Table 8:** Average results of precision, recall, and F1-score, and their weighted average for the 10 fold cross validation.

	Precision	Recall	F1-Score
Normal	1	1	1
Flooding	0.958	0.983	0.968
Scheduling	0.989	0.928	0.958
Grayhole	0.99	0.989	0.99
Blackhole	0.991	0.995	0.993
Weighted avg.	1	1	1

**Table 9:** Positive and negative rates results of the 10 fold cross validation.

	Normal	Flooding	Scheduling	Grayhole	Blackhole
TPR	1	0.98	0.93	0.99	0.99
TNR	0.98	1	1	1	1
FPR	0.02	0	0	0	0
FNR	0	0.02	0.07	0.01	0.01
Overall Accuracy	0.997				

**Table 10:** Precision, recall, and F1-score results of the holdout method.

	Precision	Recall	F1-Score
Normal	1	1	1
Flooding	0.96	0.98	0.97
Scheduling	0.99	0.93	0.96
Grayhole	0.99	0.99	0.99
Blackhole	0.99	0.99	0.99
Weighted avg.	1	1	1

**Table 11:** Comparison results of TPR for GXGBoost against the original XGBoost and other boosting classifiers models.

TPR					
	Normal	Flooding	Scheduling	Grayhole	Blackhole
AdaBoost	0.9900	0.9700	0.9000	0.8200	0.3800
GB	0.9977	0.9872	0.9239	0.8659	0.8714
XGBoost	0.9976	0.9970	0.9194	0.9409	0.9622
Proposed GXGBoost	1.0000	0.9800	0.9300	0.9900	0.9900

**Table 12:** Average execution time of classification in seconds (s).

Model	Average Classification Time
AdaBoost	10.093 s
GB	3.338 s
XGBoost	2.172 s
Proposed GXGBoost	1.905 s
